# Identification of Copy Number Variations in Xiang and Kele Pigs

**DOI:** 10.1371/journal.pone.0148565

**Published:** 2016-02-03

**Authors:** Jian Xie, Rongrong Li, Sheng Li, Xueqin Ran, Jiafu Wang, Jicai Jiang, Pengju Zhao

**Affiliations:** 1 Institute of Agro-Bioengineering and College of Life Sciences, Guizhou University, Guiyang, China; 2 College of animal Science, Guizhou University, Guiyang, China; 3 College of Animal Science and Technology, China Agricultural University, Beijing, China; Huazhong University of Science and Technology, CHINA

## Abstract

Xiang and Kele pigs are two well-known local Chinese pig breeds that possess rich genetic resources and have enormous economic and scientific value. We performed a comprehensive genomic analysis of the copy number variations (CNVs) in these breeds. CNVs are one of the most important forms of genomic variation and have profound effects on phenotypic variation. In this study, PorcineSNP60 genotyping data from 98 Xiang pigs and 22 Kele pigs were used to identify CNVs. In total, 172 candidate CNV regions (CNVRs) were identified, ranging from 3.19 kb to 8175.26 kb and covering 80.41 Mb of the pig genome. Approximately 56.40% (97/172) of the CNVRs overlapped with those identified in seven previous studies, and 43.60% (75/172) of the identified CNVRs were novel. Of the identified CNVRs, 82 (47 gain, 33 loss, and two gain-loss events that covered 4.58 Mb of the pig genome) were found only in a Xiang population with a large litter size. In contrast, 13 CNVRs (8 gain and 5 loss events) were unique to a Xiang population with small litter sizes, and 30 CNVRs (14 loss and 16 gain events) were unique to Kele pigs. The CNVRs span approximately 660 annotated *Sus scrofa* genes that are significantly enriched for specific biological functions, such as sensory perception, cognition, reproduction, ATP biosynthetic processes, and neurological processes. Many CNVR-associated genes, particularly the genes involved in reproductive traits, differed between the Xiang populations with large and small litter sizes, and these genes warrant further investigation due to their importance in determining the reproductive performance of Xiang pigs. Our results provide meaningful information about genomic variation, which may be useful in future assessments of the associations between CNVs and important phenotypes in Xiang and Kele pigs to ultimately help protect these rare breeds.

## Introduction

Pigs are economically important livestock animals that supply animal protein for human food. The pig also constitutes a reliable animal model for clinical studies of certain human diseases, including obesity and cardiovascular disease, because the anatomical and physiological characteristics of pigs are similar to those of humans [1, 2]. There are at least 16 different subspecies of pigs worldwide [3–5], mainly distributed in Europe (Yorkshire, Landrace, and Hampshire) and China (Meishan, Ningxiang, Ming, and Xiang) [5]. Due to variations in the environment, artificial selection, and feeding models, pigs have evolved into many different breeds with different phenotypic and production traits. Numerous genetic diversity studies have been conducted in pigs [6–9], and a large percentage of the genetic variations in pigs is distributed within species (intra-species) rather than between species (inter-species) [9]. Thus, intra-species genetic diversity is the most important source of diversity, and studying inter- and intra-specific genomic differences has become increasingly important.

Xiang pigs are one of the best-known local mini-pig breeds in China. They possess many advantages, including their high adaptability, crude feed tolerance, early maturation, and high levels of disease resistance. Both Xiang pigs and Kele pigs are raised in the mountainous area of Guizhou Province, which is isolated due to the difficulty in accessing this area and thus far less economically developed. Local farmers have to use traditional but inefficient methods to feed these pigs and to satisfy the low-input-with-high-returns production systems within their own farms, inevitability resulting in highly inbreeding systems [4]. These requirements have increased the genetic diversity of the breed and improved various phenotypes and traits, including growth and reproduction.

To better understand the useful variations in economically beneficial traits, several studies have evaluated variations in the genomic DNA sequences. Along with the most frequent single nucleotide polymorphism (SNP) markers and microsatellites, copy number variation (CNV) is also an important type of variation in the genome. Because a CNV region is a DNA fragment that is markedly longer than both a SNP and a microsatellite, it can cover a wider portion of the genome. Thus, a CNV may have a potentially enormous impact on the gene structure and dosage via alternating gene regulation, exposure of recessive alleles, or other mechanisms, ultimately resulting in large phenotypic effects [10, 11]. Typically, CNV refers to the insertion, deletion, amplification or more complex variations and combinations of segments of DNA ranging from 1 kilobase (Kb) to several megabases (Mb) in length compared with the reference genome. Overlapping CNVs are merged to form a copy number variation region (CNVR) [12–16]. Previous studies in humans [17–23], cattle [10, 13, 15], pandas [16], sheep [12, 14], chickens [24], horses [25], and pigs [3, 26–30] have indicated that CNVs can affect gene expression, phenotypic variation, disease susceptibility, and adaptation by rearranging genes and altering gene dosage [24, 26]. For instance, the human *PLP1* gene is altered in patients with spastic paraplegia type 2 [31]; a duplication of intron 1 of *SOX5* causes the Pea-comb phenotype in chickens [32]; a 4.6-kb intronic duplication of *STX17* results in hair graying in horses [33]; and a duplication of *KIT* is associated with a white coat in pigs [34]. Thus, CNVs have become a new genetic marker with great promise for use in genetics research [26].

At present, two methodologies are commonly used to assess CNVs: Array Comparative Genomic Hybridization (aCGH) and SNP Genotyping Chips (Illumina or Affymetrix). aCGH is a molecular genetics approach based on fluorescence *in situ* hybridization (FISH) and highly sensitive, highly accurate chip technology [27, 35, 36]. In contrast, Whole-Genome SNP Genotyping BeadChips provide an alternative, more desirable method for genome-wide association studies (GWAS) and CNV identification [30, 37]. The PorcineSNP60K Genotyping BeadChip was designed using a high-density array that features 64,232 SNPs, providing uniform, genome-wide coverage with an average spacing of 43.4 kb.

Many studies have evaluated the structure and function of CNVs in livestock, such as cattle, horses, dogs, pigs, sheep, and chickens [13, 14, 24, 29, 38]. Surveys of CNVs in pigs have also been conducted using SNP chips or aCGH. For example, in previous studies, 37 CNVRs were identified in 12 unrelated Duroc boars [27], 49 CNVRs were detected in 55 animals from an Iberian x Landrace cross (IBMAP), 259 CNVRs were identified in 12 pigs from different pig breeds [3], and 382 CNVRs across the genome were identified in three pure breeds (Yorkshire, Landrace, and Songliao Black) and one Duroc x Erhualian crossbreed population [26]. Additionally, 105 CNVRs have been identified in 96 Chinese native Dahe pigs, Tibetan pigs and Wuzhishan pigs [39]. Previous studies have indicated that CNVs vary enormously between different species and display diversity even within populations. However, though many CNVRs have been identified in some pig breeds, including Duroc, Yorkshire, Landrace, and Erhualian, no reports on CNVs in the Xiang and Kele breeds are currently available.

The objectives of this study were to comprehensively interrogate and characterize CNVs across the Xiang and Kele genomes to identify the relationships between CNVRs and quantitative traits, to assess the genetic diversity between or within populations, and to compare the CNVs in Xiang and Kele pigs with those of other previously studied breeds.

## Results

### Genome-wide CNV detection

**Fig 1:**
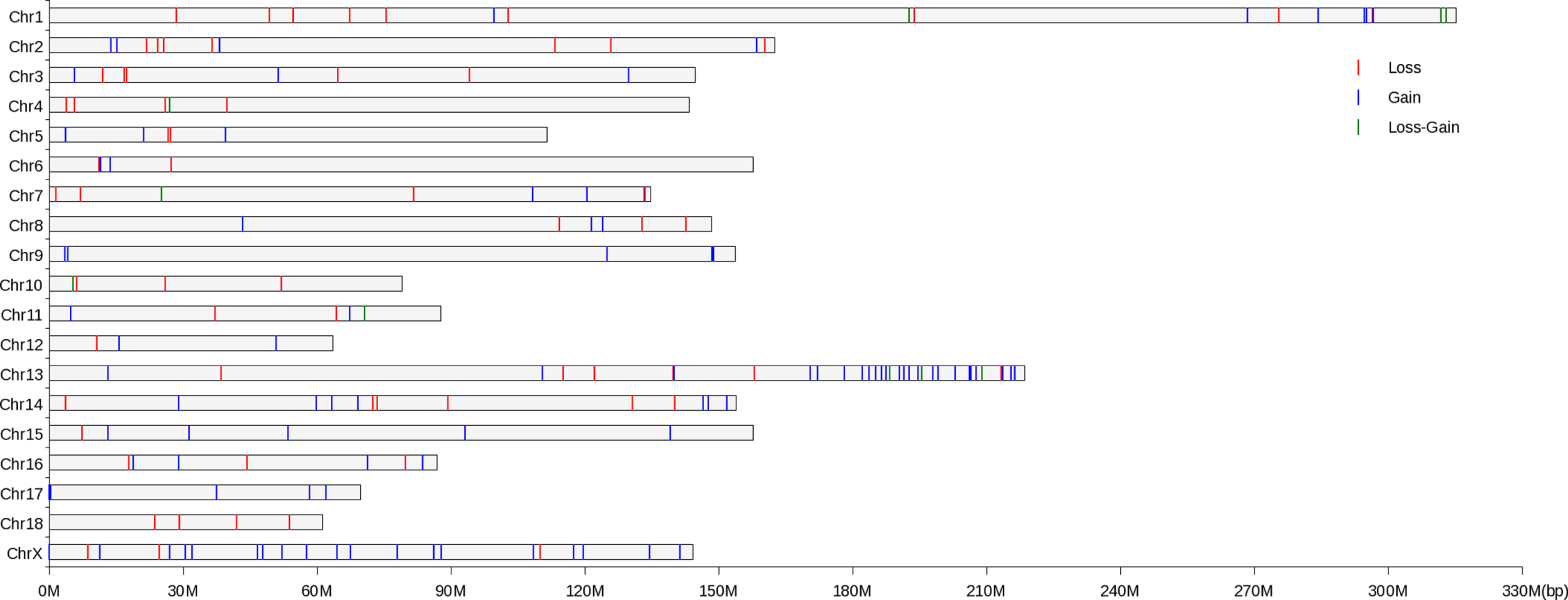
Distribution of detected CNVRs in Xiang and Kele pigs. The X-axis values represent the chromosome position in Mb, based on the *Sus scrofa* 10.2 reference genome assembly. The Y-axis values show the chromosome number.

In the present study, 98 Xiang pigs and 22 Kele pigs were evaluated using the Illumina PorcineSNP60K BeadChip with strict quality control measures for CNV identification. Overall, using PennCNV, 401 CNVs were identified on 18 pairs of autosomes, and 274 CNVs were assessed on the X chromosome. The average number of CNVs was 5.63 per individual. By aggregating the overlapping CNVs, a total of 150 CNVRs on the autosomes and 22 CNVRs on the X chromosome were identified, with a total coverage of 80.41 Mb corresponding to 2.98% of the pig genome. The length of these CNVRs ranged from 3.19 kb to 8175.26 kb, with a mean of 467.53 kb and a median of 123.39 kb. Of the 172 CNVRs, 97 (56.40%) CNVRs were called as gain events, 65 (37.80%) were called as loss events, and the remaining 10 CNVRs (5.81%) were called as gain-loss events (gain and loss within the same region). The 172 CNVRs were not uniformly distributed on the 18 autosomes and the X chromosome (Fig 1). On average, 1.43 CNVRs were identified per individual. Thirty-three (19.19%) CNVRs (maximum) were identified on *Sus scrofa* chromosome (SSC) 13, and three (1.74%) CNVRs (minimum) were found on SSC 12. The length of the CNVRs on each chromosome ranged from 0.25 Mb (SSC 12) to 37.99 Mb (SSC X). Moreover, the numbers of CNVRs distributed in individuals differed; 12 individuals only had one identified CNVR, 22 individuals had two identified CNVRs, and 82 individuals had more than three identified CNVRs. Overall, 26, 18, 13, 12, and 12 CNVRs were identified from samples of 1X08F1, 1X10, 1X09F1, 1X87F1, and 1X68, respectively (Table A in S1 File).

### Gene content and functional analysis

The Ensembl Genes 80 Database as provided in the BioMart data management system (http://asia.ensembl.org/biomart/martview/) was queried to retrieve the gene content. Overall, 660 Ensembl genes that completely or partially overlapped with the identified CNVRs were retrieved, including 508 (76.97%) protein-coding genes, 76 (11.51%) pseudogenes, 11 (1.67%) snoRNAs, 25 (3.79%) miRNAs, 28 (4.24%) snRNAs, three misc-RNAs, two non-coding genes, five processed transcripts, one antisense sequence, and one lincRNA (Table B in S1 File). These genes were distributed throughout 103 (59.88%) CNVRs. Sixty-three (36.62%) CNVRs contained or overlapped with at least two genes, and only one gene was retrieved from 40 (23.25%) CNVRs. We could not retrieve any gene information for the other CNVRs. The densest gene region (59 genes) corresponded to CNVR-163, which is located on SSC X. CNVR-28 contained 49 genes and is located on SSC 2, whereas CNVR-54 contained 46 genes and is located on SSC 7. Of the 660 genes, 491 were identified as gain events, 52 as loss events, and 117 as gain-loss events.

Gene Ontology (GO) annotations and Kyoto Encyclopedia of Genes and Genomes (KEGG) pathway analysis were performed using the DAVID bioinformatics resources (http://david.abcc.ncifcrf.gov/) to gain insight into the functional enrichment of the CNVs. Because the pig genome annotation is not complete, we first converted the pig Ensembl gene IDs to homologous human Ensembl gene IDs in BioMart, then set all of the human genes that have homologs in the pig genome as background, and finally carried out the GO and pathway analysis. Gene Ontology analysis showed that 341 genes were enriched with 34 statistically significant GO terms (*P*< 0.05; Table C in S1 File). The relevant GO terms were mainly associated with sensory perception, olfactory receptor activity, cognition, G-protein-coupled receptor protein signaling pathways, gonad development, cell surface receptor-linked signal transduction, synaptic vesicle endocytosis, developmental maturation, and neurological processes (Table 1). KEGG pathway analysis indicated that the genes within the CNVRs were involved in olfactory transduction, oxidative phosphorylation and ABC transporters (Table D in S1 File). To reveal the relationships between the CNVRs and QTLs, we analyzed the overlap between the identified CNVRs and the QTL regions collected in the pig QTL database (http://www.animalgenome.org/cgi-bin/QTLdb/SS/index). The current release of the pig QTLdb contains 12,618 QTLs representing 656 different traits (2015, 2, 1). The analysis showed that 172 CNVRs encompassed or overlapped with 7676 pig QTLs, which affect many traits, such as reproduction, production, meat and carcass quality, health, and growth (Table E in S1 File).

**Table 1:** Enriched Gene Ontology (GO) terms identified in this study.

Term	Count	Pvalue	Benjamini
GO:0007608~sensory perception of smell	52	9.55E-34	1.39E-30
GO:0007606~sensory perception of chemical stimulus	52	7.23E-32	7.23E-32
GO:0050890~cognition	60	1.44E-22	7.03E-20
GO:0007600~sensory perception	56	1.85E-22	6.76E-20
GO:0007186~G-protein-coupled receptor protein signaling pathway	64	4.81E-21	1.41E-18
GO:0050877~neurological system process	66	2.06E-19	5.02E-17
GO:0007166~cell surface receptor linked signal transduction	73	1.76E-13	3.67E-11
GO:0048488~synaptic vesicle endocytosis	3	0.023814054	0.98774306
GO:0021700~developmental maturation	6	0.03500806	0.99692618
GO:0008406~gonad development	6	0.047818524	0.999222049
GO:0009152~purine ribonucleotide biosynthetic process	6	0.047818524	0.999222049

### Comparison with other CNV studies

We compared the CNVRs identified in the present study with the results of seven previous studies (Table 2) in terms of the different sizes and structures of the pig populations investigated as well as the platforms and algorithms used for CNV detection (Table F in S1 File). Thirty-three of the 172 CNVRs overlapped with the results reported by Wang *et al*. (2014), 17 CNVRs overlapped with the findings obtained by Wang *et al*. (2013), 11 CNVRs overlapped with the results found by Wang *et al*. (2012), 52 CNVRs overlapped with the CNVRs identified by Wang *et al*. (2014), 10 CNVRs overlapped with those reported by Fernández *et al*. (2014), 14 CNVRs overlapped with the results reported by Schiavo *et al*. (2014), and 39 CNVRs overlapped with those identified by Dong *et al*. (2015). In total, 97 (56.40%) CNVRs either overlapped with or were identical to those identified in seven previous studies. The remaining 75 (43.60%) CNVRs were likely novel (Table G in S1 File). The greatest overlap (30.23% CNVR count, 31.78% CNVR length) was found with the results reported by Wang *et al*. (2014). These researchers used the PorcineSNP60K BeadChip and PennCNV algorithm to detect 348 CNV regions in 302 individuals from six Chinese indigenous breeds (Laiwu, Wuzhishan, Luchuan, Tongcheng, Ningxiang, and Bama pigs), three Western breeds (Landrace, Duroc, and Yorkshire), and one hybrid breed (Tongcheng x Duroc). The second highest overlap in the CNVR count (22.67%) and CNVR length (16.12%) was obtained with the results reported by Dong *et al*. (2015), who used the PorcineSNP60K BeadChip and PennCNV algorithms to identify 105 CNV regions in 98 individuals from Tibetan pigs, Dahe pigs, and Wuzhishan pigs. Less overlap was observed between our results and those obtained by Schiavo *et al*. (2014), Fernández *et al*. (2014), and Wang *et al*. (2012, 2013).

**Table 2:** Comparison of CNVRs identified in the current study with previously reported porcine CNVRs.

Study	Sample size	CNVRs	Overlapping	Platform
Current study	120	172		BeadChip
Jiying Wang *et al*.(2013)	14	63	17	BeadChip
Jiying Wang *et al*.(2012)	474	382	11	BeadChip
Yana Wang *et al*. (2014)	302	348	52	BeadChip
Fernández A I *et al*.(2014)	223	65	10	BeadChip
G. Schiavo *et al*.(2014)	297	170	14	BeadChip
K. Dong *et al*.(2015)	98	105	39	BeadChip
Wang Jiying *et al*.(2014)	12	1344	33	aCGH

### Differences in the CNVRs between Xiang pigs and Kele pigs

There are many differences between the two breeds (Xiang and Kele) evaluated in this study. Ninety-five CNVRs (55 gain, 38 loss, and two gain-loss events) were specifically identified in the Xiang breed, whereas 30 CNVRs (16 gain and 14 loss events) were found only in Kele pigs (Table H in S1 File). An average of 0.97 CNVRs were obtained for Xiang pigs, whereas an average of 1.07 CNVRs were obtained for Kele pigs. CNVRs were detected on all chromosomes in Xiang pigs, whereas in Kele pigs, no CNVRs were found on SSC 6, SSC 8, SSC 17, or SSC X. A total of 327Ensembl genes were encompassed by or partially overlapped with the CNVRs of Xiang pigs. These genes included 235 protein-coding genes, 40 pseudogenes, 19 snRNAs, 18 miRNAs, and nine snoRNAs. In contrast, only 44 unique Ensembl genes were retrieved in Kele pigs. These included 41 protein-coding genes, one pseudogene, one misc-RNA, and one processed transcript (Table I in S1 File). GO analysis of the CNVR-associated genes identified in both Xiang and Kele pigs showed that these genes were involved in olfactory receptor activity, cell surface receptors, the plasma membrane, and G-protein coupled receptor protein signaling pathways. Analysis of the differences between the breeds revealed that the CNVR-associated genes in Xiang pigs were involved in reproduction, cell morphogenesis, ribonucleotide metabolism, cellular ion homeostasis, ATP biosynthetic processes, and superoxide response, whereas the CNVR-associated genes in Kele pigs were involved in phagocytosis and regulation of protein modification processes.

**Fig 2:**
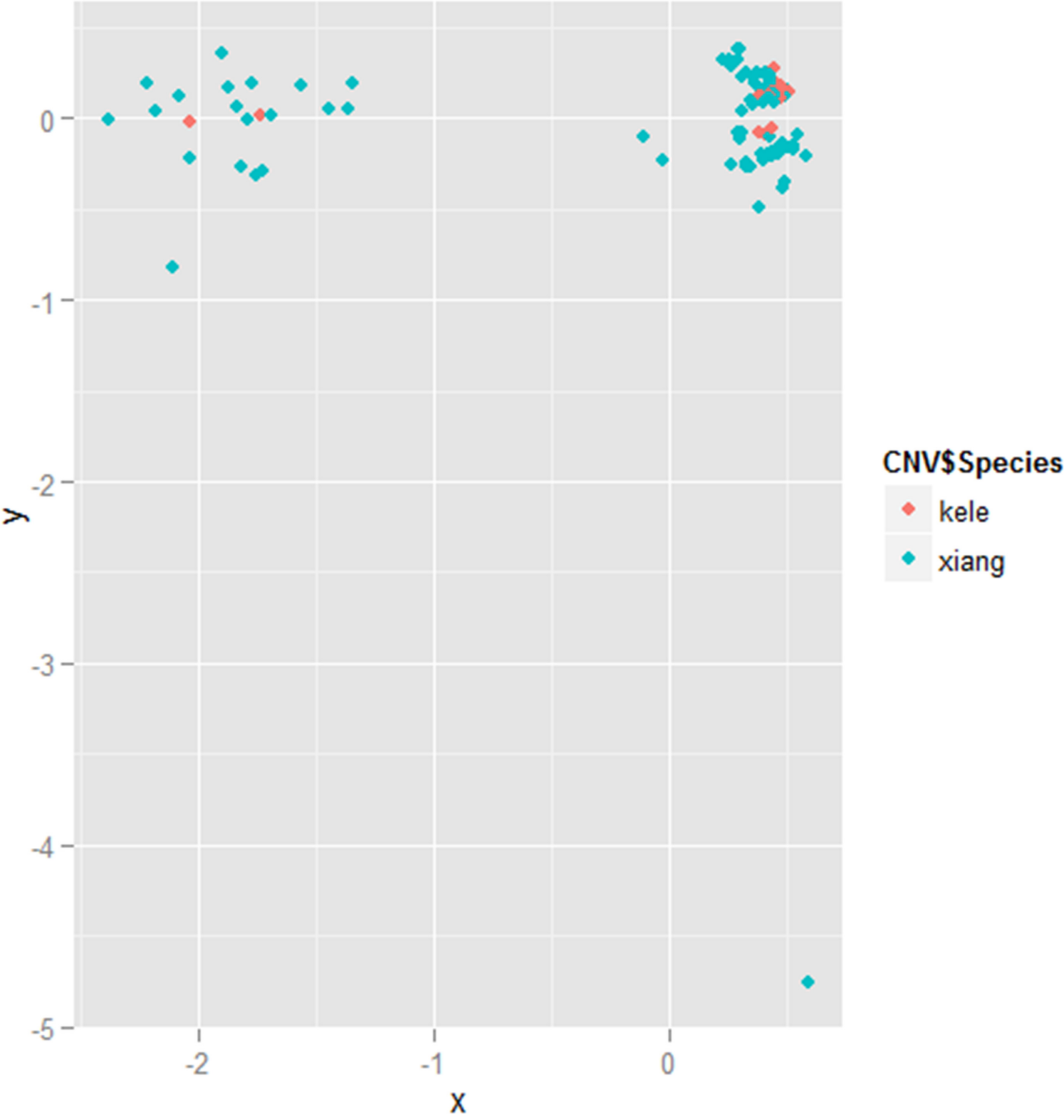
Hierarchical clustering analysis based on all available CNVR information. Individuals were plotted according to their coordinates on the biplot of x versus y. Blue: Xiang breed, Red: Kele breed.

To investigate the differences in the distribution patterns between the two pig breeds, we performed a hierarchical clustering analysis using R software (Fig 2). All 117 samples were obviously clustered into two categories; 95 samples (81.19%) were in the first group and 21 were (17.95%) in the second group (left and right, respectively, in Fig 2).

### Variations within the Xiang pig population

The distribution of CNVs also revealed wide variation in Xiang pigs at the population level. According to the original sampling records, we found 82 CNVRs in the population of Xiang pigs with large litter sizes (in which multiparous sows can farrow at least 12 piglets), including 47 gain, 33 loss, and two gain-loss events that covered 45.84 Mb of the pig genome. Moreover, 13 CNVRs in the Xiang population with small litter sizes (in which multiparous sows can farrow at most eight piglets) included eight gain and five loss events that covered 1.22 Mb of the pig genome (Table H in S1 File). The CNV-associated genes also varied between these populations: 320 genes (Table J in S1 File) were only retrieved from the large litter size population; these genes were mainly involved in olfactory receptors, cell adhesion, trans-membrane proteins, reproduction, cell cycle regulation, purine and pyrimidine nucleotide metabolic processes, ATP biosynthetic processes, and skeletal muscle tissue development, whereas only seven genes (Table K in S1 File) were retrieved from the small litter size population. Notably, the *ADAMTS-1*, *AR*, *KIT*, *MED12*, *NR0B1*, *PN-1*, and *SOD1* genes, which are involved in reproductive traits, were only found in the Xiang pig population with large litter sizes. In this population, the CNV-associated genes retrieved that might play an important role in reproductive traits included *ADAMTS-1* (*A disintegrin and metallo-proteinase with thrombospondin motifs 1*), which is involved in normal folliculogenesis and the ovulatory process [40–42], and *AR* (*androgen receptor*), which is essential for normal fertility and mediates fetal sexual differentiation and pubertal sexual maturation [43]. Additionally, KIT is a receptor encoded by the *KIT* gene that is present on oocytes and theca cells and functions as a regulator of oogenesis and folliculogenesis [44, 45]. Due to the potential functions of the CNV-associated genes, we hypothesized that the CNVs might affect different reproductive traits in Xiang pigs.

### Validation of CNVs by qPCR

Twenty-six CNVRs were randomly selected from the 172 CNVRs detected using the PorcineSNP60K BeadChip for validation via quantitative PCR (qPCR). These 26 CNVRs ranged from 10.44 kb to 8175.26 kb and presented different statuses (13 gain, six loss, and seven gain-loss events). Sixteen of the selected CNVRs (61.54%) were validated by qPCR (Table L in S1 File). Four CNVRs (CNVR-24, CNVR-35, CNVR-50, and CNVR-106) and approximately 150 samples were selected for further validation of the CNVRs. CNVR-35 was validated in 110 out of 157 samples, CNVR-50 was validated in 93 out of 137, CNVR-24 was validated in 70 out of 135 samples, and CNVR-106 was validated in 92 out of 97 samples.

## Discussion

One hundred seventy-two CNVRs were identified on autosomes and the X chromosome; these elements covered 80.41 Mb of the pig genome and corresponded to 2.98% of the pig genome sequence. This result was consistent with those of previous studies in pigs [3, 29, 30, 36]. The lengths of the CNVRs ranged from 3.19 to 8175.26 kb, with a mean of 467.52 kb and a median of 123.39 kb. We found that 97 (56.40%) of the CNVRs found in the present study overlapped with those found in seven previous studies that used the PorcineSNP60 BeadChip or aCGH to identify CNVs. While more than half of the CNVRs identified in this study could be validated by previous studies, approximately 43.60% of the CNVRs had not been previously reported and were therefore likely novel, which suggests that CNV detection in pigs is far from saturated [39]. The differences in the overlap rate among these studies could be due to differences in many factors, including sample sizes, sample genetic backgrounds, detection platforms, and CNV calling algorithms. Of the CNVRs identified in our study, 52 and 39 overlapped with the results reported by Wang *et al*. (2014) and Dong *et al*. (2015), respectively; both of these previous studies primarily involved indigenous Chinese breeds such as Bama Xiang pigs, Wuzhishan pigs, and Tibetan pigs, which, along with Xiang pigs, are characteristic Chinese breeds with similar genetic backgrounds. Different breeds share different amounts of CNVRs, and more shared CNVRs indicates a closer genetic relationship [46]. Our study provided the first identification of a set of CNVRs in Xiang pigs and found a large number of new, breed-specific CNVRs. Xiang pigs are raised in the Eastern Guizhou mountainous area, which has extremely challenging travel conditions, a poor economy and a lower level of access to science and technology. Under such conditions, high levels of inbreeding are inevitable, and this situation may be responsible for the identification of CNVs that are not found in other breeds. Additionally, SNP BeadChips and CGH arrays detect different CNVs due to differences in the calling techniques, genome coverage and resolution between these methods. Genomic waves have been described as the existence of wave patterns in genomic data across all chromosomes, which may lower the accuracy of the annotation of DNA variants such as CNVs [47]. Thus, *gc*- correction was applied to eliminate genomic waving in this study.

In this study, a total of 58 CNVRs were detected in Kele pigs, of which 30 were specific to the Kele breed and 28 were shared with the Xiang breed. The 28 CNVRs common to the two pig breeds represented approximately 16.3% of all CNVRs found in the two breeds (28/172). A similar pattern has been found in other laboratories. There are many common CNVRs among Chinese pig breeds, and some of these are shared between Chinese and European pig breeds [36, 39, 48, 49].The large fractions of common CNVRs between different pig breeds suggest that the majority of these variations are likely to be neutral or nearly neutral and may not influence the diversity of pig breeds [49]. The large number of common CNVs in Chinese native pig breeds might also be attributable to the fact that all of the Chinese native pig breeds were domesticated from one or several Asian wild boars approximately 10,000 years ago [50]. However, many CNVs are specific to a single Chinese pig breed. These unique CNVs may arise from environmental and artificial selective pressures.

Both inter-species and intra-species differences in CNVs were detected. Xiang pigs are a special and precious indigenous breed with a unique genetic background. Local farmers care for their pigs relatively independently, resulting in little genetic interchange among these Xiang pig populations. The living environment and feeding conditions result in rich genetic and phenotypic diversity among Xiang pigs, particularly with respect to growth and reproduction. The distribution of CNVs identified in this study was widely variable within Xiang pigs. A high number of Xiang pig-specific genes, with 82 CNVRs (47 gain, 33 loss, and two gain-loss events) containing 320 Ensembl genes, were retrieved from the large-litter-size population, whereas only a few specific genes, with 13 CNVRs (seven gain and five loss events) containing seven Ensembl genes, were retrieved from the small-litter-size group. Few individuals with an elevated number of CNVRs were detected. If an individual possesses too many CNVs, gene dosage effects and gene rearrangement are inevitable, if these gain or loss events are located in certain regions, such as in functional genes related to survival, they might impact phenotype and development and even lead to death. Consistent with this perspective, very few individuals with many CNVs are detected, indicating that most animals can survive only when a limited number of CNVs are present in the genome [36]. The influence of CNVRs on phenotypic variations was also obvious from the data. Interestingly, an analysis of the function of the CNV-associated genes identified in Xiang pigs highlighted the genes *ADAMTS-1*, *AR*, *KIT*, *MED12*, *NR0B1*, *PN-1*, and *SOD1*, which are known to be involved in reproductive traits, in the population with large litter sizes. *ADAMTS-1* is induced in granulosa cells by LH during the periovulatory period and may be regulated by the progesterone receptor in the hormonal pathway leading to ovulation. A lack of *ADAMTS-1* results in a decrease in female fertility and could influence human infertility [40–42, 51]. Androgen Receptor (AR) plays an important role in male reproduction. Androgens regulate pubertal sexual maturation and fetal sexual differentiation and maintain post-pubertal virility [43]. CNVR-60 included the *KIT* gene, consistent with the results of previous studies [30, 36, 37, 39]. A duplication, triplication, or splice mutation in *KIT* results in the white coat phenotype in pigs [34]. Moreover, *KIT* regulates oogenesis and folliculogenesis [44, 45]. *NR0B1*, which encodes a 470-amino-acid protein containing a DNA-binding domain that is mediated by the retinoic acid receptor, is expressed in developmentally regulated patterns at all levels of the hypothalamic-pituitary-adrenal-gonadal axis, including both the ovaries and the testes [52]. The deletion of *NR0B1* causes congenital adrenal hypoplasia, whereas its duplication leads to a pseudo-female phenotype and gonadal dysgenesis [53]. *PN-1* belongs to the serpin superfamily and regulates the proteolytic activity of thrombin, plasminogen activators, trypsin and plasmin; it is expressed in many tissues, particularly adult seminal vesicles [54, 55]. In addition, *SOD1* eliminates superoxide generated during steroidogenesis and may play a role in mediating follicular development, ovulation, and luteal functions [56]. We infer that differences in copy number variation may result in differences in phenotypes, such as the litter size trait, between populations. Because the genes identified in this study may influence reproductive traits, further studies should be performed to investigate the structures and potential functions of these CNV-associated genes.

Six hundred sixty annotated genes were associated with the 172 identified CNVRs. SSC 4, 10, 11, and 12 each harbored only one gene, whereas SSC 1, 2, 7, 13 and X contained 51, 68, 68, 128, and 268 genes, respectively. The distribution of CNVRs among the chromosomes demonstrated that the number of SNPs and the lengths of the chromosomes may have affected the CNVR mapping; longer chromosomes may, of course, contain more and larger CNVRs, although many other factors may also affect the CNVR distribution [28, 57]. Previous studies have indicated that some genomic regions are particularly susceptible to structural rearrangements that generate CNV hotspots [58]. CNVR-163, CNVR-28, CNVR-54, CNVR-160, CNVR-164, and CNVR-17 contain 59, 49, 46, 39, 29 and 29 genes, respectively. Only one gene was retrieved for forty different CNVRs, and no gene information was retrieved for 69 other CNVRs. These results demonstrate that CNVs are commonly located in gene-poor regions [59, 60]. This situation may occur because duplications or deletions of large DNA segments may have more deleterious effects in gene-rich regions, leading to likely natural selection against individuals harboring these types of changes. In contrast, many genes were retrieved for the detected CNVRs, suggesting that the Illumina PorcineSNP60K BeadChip may be biased toward gene-rich regions [61]. Of the CNVRs evaluated in this study, 52 genes were loss events, 491 were gain events, and 117 were gain-loss events. GO analysis indicated that the genes retrieved in gain CNVRs are involved in sensory perception, neurological system process, G-protein-coupled receptor protein signaling pathway and cell surface receptor-linked signal transduction, whereas genes in the loss events are involved in olfactory receptor activity (Table C in S1 File). Most of these genes participated in the olfactory receptor activity process, which suggests that some of the olfactory receptor variations observed in pigs may be basic physiological requirements for adapting to their specific living conditions, such as scavenging for food [50, 62, 63].

Functional analyses, such as GO and KEGG, were performed to gain insight into the functional enrichment of the CNVs. Because the porcine gene annotation is far from complete, annotations of homologous genes from humans were used in this analysis. We found that the genes significantly enriched in the identified CNVs were involved in sensory perception, olfactory receptor activity, cognition, developmental maturation, gonad development, neurological processes, G-protein-coupled receptor protein signaling pathways, and cell surface receptor-linked signal transduction. It is particularly interesting to note that some of the CNVR-associated genes that were only retrieved from the Xiang population with large litter sizes were involved in reproduction, which may imply that some CNVRs were related to the reproductive performance of Xiang pigs. Previous studies have shown that CNVs play an important role in phenotypic variation. An analysis of the overlap between CNVRs and reported QTL regions demonstrated that the CNVRs detected in this study are mainly involved in reproductive traits, production traits, meat and carcass quality traits, health traits, and growth traits. This result is consistent with the findings from the GO analysis. These preliminary analyses provide promising results, indicating that further studies should be performed.

To confirm these potential CNVRs, 26 CNVRs (13 gain, 6 six loss and seven gain-loss events) were randomly selected for validation by real-time quantitative PCR, and a total of 16 CNVRs (61.54%) were validated. This validation rate is high compared with those in many previous studies [3, 10, 13, 33, 38] and lower than those in a few recent studies [18, 30]. Although this validation rate seems poor, it should be noted that qPCR is not trivial for a highly error-prone preliminary genome assembly. Several factors may have contributed to the discrepancy in the number of CNVRs detected by the PennCNV and qPCR methods [27, 64]. First, the *Sus scrofa* 10.2 assembly is far from complete, and the Porcine SNP60K BeadChip has a lower probe density. Thus, the identified CNVRs may appear larger than they are. Therefore, the primers may have been designed based on sequences located outside of the CNVRs. Second, compared with the reference genome assembly, SNPs and small indels in the CNVRs may have also affected the qPCR results [29].

## Conclusions

In this study, we used PorcineSNP60K genotyping data to reveal the distribution of an unprecedented number of 172 CNVRs from 120 individuals. These CNVRs were non-randomly distributed in the porcine genome. Moreover, 56.40% of the CNVRs identified in the present study were identical to those found in previous studies, whereas 43.60% were likely novel, suggesting that CNV detection in pigs is far from saturated. Notably, we found a high degree of CNVR diversity within Xiang pigs, demonstrating that copy number variation may be an important source of phenotypic diversity within this breed. Functional annotation illustrated that these CNVRs and CNVR-associated genes are involved in a variety of molecular functions and may play important roles in relevant traits, including reproductive traits. The results of our study provide meaningful genomic variation information that may benefit future studies that address the associations between CNVs and important phenotypes in Xiang pigs and aim to protect this precious breed.

## Materials and Methods

### Ethics Statement

Whole-blood samples were drawn from 120 pigs in accordance with the guidelines approved by the Biological Studies Animal Care and Use Committee of Guizhou Province for animal experiments. All efforts were made to minimize any discomfort during blood collection.

### Samples

Ninety-eight Xiang pigs and 22 Kele pigs, including 105 sows and 15 boars, were selected for this study. First, 5 ml of whole blood was drawn from the precaval vein of each pig, and the bleeding was stopped immediately. Genomic DNA was extracted using a TIANamp Blood DNA Kit and then analyzed by spectrophotometry and agarose gel electrophoresis.

### CNV calling and statistical analysis

All 120 samples were genotyped using a Porcine SNP60K BeadChip, and the raw data were extracted using GenomeStudio (Version 1.9.4, Illumina, Inc.). We used a strict quality control threshold (call rate > 98%, call frequency > 90%) to minimize the false-positive rate and eliminate low-quality samples. After quality control, no samples were excluded from CNV detection. The PennCNV software using a hidden Markov model (HMM), which allows detection of CNVs from Illumina or Affymetrix SNP chip data, was employed for CNV identification. PennCNV integrates various sets of information, including the total signal intensity of the log R Ratio (LRR), the distance between neighboring SNPs, the B allele frequency (BAF), the population frequency of the B allele (PFB) of SNPs, pedigree information, and the–*gc* model to adjust for “genomic waves” [11, 43, 65, 66]. We could not obtain pedigree information for many of the samples because the samples were obtained from multiple locations across Congjiang County, China. Thus, pedigree information was not incorporated into the analysis. Moreover, the CNVs were aggregated using CNVRuler software [67].

### Hierarchical clustering analysis

A hierarchical clustering analysis for all test samples was performed according to their CNV similarities. We first converted the copy number data to a scoring matrix for each individual. If there was no CNVR at the locus, we encoded a value of “0” for the locus; otherwise, if there was CNVR present at the locus (either gain or loss), we encoded a value of “1”. Hierarchical clustering (unweighted pair-group method with arithmetic means) was performed on the matrix composed of the individual vectors using the “hclust” function in R (www.r-project.org) [68–71].

### Functional enrichment analysis of CNVRs

Gene information was retrieved from the BioMart data management system (http://asia.ensembl.org/biomart/martview/). To obtain insight into whether genes overlapping or within CNVRs exert certain functions, Gene Ontology (GO) annotation and Kyoto Encyclopedia of Genes and Genomes (KEGG) pathway analyses were performed using the online software DAVID (http://david.abcc.ncifcrf.gov/).

### qPCR confirmation

Quantitative PCR (qPCR) was performed to validate 26 CNVRs randomly selected from the 172 CNVRs identified by PennCNV. Nucleotide similarity searches of the GenBank database using blastn showed that the glucagon gene (*GCG*), which has been shown to be present in a single copy in many different species [72, 73], is highly conserved. Therefore, *GCG* was selected as a control. The primers were designed using Primer 5.0. Before validating the CNVRs, the primers were confirmed to have PCR efficiencies ranging from 95% to 110%. All of the qPCR reactions were run on a BIO-RAD CFX98^TM^ Real-Time System according to the manufacturer’s instructions and recommended cycling conditions. Each sample was tested in triplicate reactions using a 10-μl volume containing 5 μl of 2x Super Real Pre Mix Plus, 0.3 μl of each forward and reverse primer (10 pM/μl) and 1 μl of 20 ng/μl genomic DNA. The qPCR conditions were as follows: initial denaturation at 95°C for 3 min followed by 40 cycles of denaturation at 94°C for 10 s and combined annealing and extension at 58°C for 30 s.

## Supporting Information

S1 File Supporting tables. CNVRs detected in the study (Table A). Information of genes in the identified CNVRs (Table B). Gene ontology (GO) analysis of genes in identified CNVRs (Table C). Pathway analysis of genes in identified CNVRs (Table D). Previously reported QTLs overlapping with identified CNVRs (Table E). Comparison between identified CNVRs and those reported in other pig CNVR papers (Table F). Special CNVRs and gene information in this study compare with other studies (Table G). Information of CNVRs in different populations (Table H). Information of genes in the CNVRs of large litter size Xiang pig (Table I). Information of genes in the CNVRs of small litter size Xiang pig (Table J). Information of genes in the CNVRs of Kele pig (Table K). Information and the primers used in qPCR analysis of the 26 CNVRs chosen to be validated (Table L).
(XLSX)
